# Relevance of Competency Based Education for Architectural Education in India

**DOI:** 10.12688/f1000research.148617.2

**Published:** 2024-11-11

**Authors:** Nikhil Kohale, Pradeep Kini, Ciraj Mohammed

**Affiliations:** 1 Manipal School of Architecture and Planning, Manipal Academy of Higher Education, Karnataka, Manipal, 576104, India; 2 College of Medicine & Health Sciences, National University of Science & Technology, National University of Science & Technology, Oman, P.O. 391, P.C. 321, Oman

**Keywords:** Quality, Value, Life-long learning, Assessment, Knowledge integration, Accountability, Architectural education, Affective domain, Curriculum, Proficiency.

## Abstract

**Background:**

A holistic architectural education is the culmination of learning knowledge, skills, attitudes, and values, which eventually reflects in the quality of graduates. Even though different schools of thought have made various kinds of qualitative contributions towards the evolution of architectural education in India, it has largely been dominated by the quantitative and technical aspects of its regulating framework. Architects engage with the demanding contradictions between responsibilities of an ethical nature, the dynamic challenges of practice, and the intricacies of architectural imagination. The aesthetical and imaginative foundations of the field make it incumbent upon the architects to possess a balance of ecumenical proficiencies for accountability and personalization. The purpose of the study is to identify relevant attributes of Competency Based Education (CBE) that can be adopted for architectural education in India.

**Methods:**

This research follows a narrative review approach and a descriptive-analytic method to broadly understand the attributes of CBE and its potential relevance to architectural education in India. 323 articles were searched on various search strings based on their relevance to the inquiry. 76 documents written in English language were included and appraised through the Scale of the Assessment for Narrative Review Articles (SANRA) tool to avoid any risk of bias. The PRISMA 2020 checklist and flow diagram has been used to report the findings of this narrative review.

**Result:**

The study identifies eight critical parameters of CBE with respect to its definitions, origins, transitions, regulatory environment, characteristics, approaches and implications on teaching-learning, frameworks and models of assessment; and challenges, which makes a case for the relevance of CBE for architectural education in India, which hasn’t been explored yet.

**Conclusion:**

The broader expectations of ‘being competent’ can be addressed through a conscious adoption of strategies of relevant attributes of CBE which can encourage building attitudes and temperament for life-long learning.

## Introduction

Architectural education at its core, is a culmination of learning knowledge, skills, attitudes as well as values which are reflective of the challenges and possibilities of a genuine human experience, which eventually reflects in the quality of graduates. The evolution of architecture and its education, is an important professional strand of our environmental fabric as it is reflective of the possibilities of a genuine human experience, which eventually reflects the quality of graduates. As per the Charter for Architectural Education framed by the International Union of Architects (UNESCO, UIA, 2008), the main goal of architectural education should be to produce professionals who are intellectually mature, competent, ecologically conscious, socially responsible, and excellent world citizens.

In a globalized world, mobility between countries is increasingly desired, laying implicit expectations on the quality of education to be at least comparable, if not at par. The majority of professionals that one meets frequently lament that the average quality of fresh graduates they hire is drastically below what is required ( Khan & Khan, 2019; Chandavarkar, 2018; Menon, 2017; Dua and chahal, 2014; Mehta, 2006). Such concerns highlight the questions on the qualitative aspects surrounding assessment of quality in learning of the graduates. While enough attention is given to various attributes which constitutes a technical architectural education, there is a need to define competence as an explicitly holistic part of the learning framework for assessment of learner’s outcomes. Assessment should not just be the driver, but it can also be a motivator for learning. The central research question of this study is to explore and identify the core aspects of Competency Based Education (CBE) are relevant for architectural education in India.

CBE, as a formal process, is relatively new and not yet a widely practiced approach in the discipline of architecture in India. Citing differences between integral aspects of *‘thinking’* and *‘doing’* from a regulatory point of view, Prof. A. G. K. Menon (2017) points how vocationalising can create a divide between the two equally important aspects of the domain. With that rationale, he advocates the need to delink the licensing of architects from the process of education, to ensure quality of architects for practice, which requires specific competences. This is the norm in all developed countries and for the certification of this knowledge new systems must be developed ( Menon, 2017). CBE has been explored in different capacities in different countries for a wide range of programs ( Barman & Konwar, 2011; Bohain, 2018; Bernikova, 2017; Biemans, et al., 2004; Book, 2014; Ford, 2014; Nodine, 2016). The article presents the findings of a narrative literature review and identifies attributes of a CBE which makes it relevant for the education of architects in India. In its intent, the study also makes a case of how such steps can provide the initial foundations on bridging gaps in proficiency and quality when compared across the continents to produce world citizens in an effort to provide quality education for all.

Even though competency of an individual can be holistically gauged or assessed from the demonstration in a practice environment, its orientation and inculcation need to begin in the educational environment. The concerns related to architecture are contextual in nature and are more about the ability to problematize them so that relevant solutions could be sought. This requires an approach towards encouraging critical thinking and developing one’s abilities in a holistic manner. While content-based education often relies upon disciplinary knowledge alone, in a world of growing complexities of multi-disciplinary nature there is a need to embrace learning which can enhance mastery in an effort to develop competencies to overcome such challenges ( Tahirsylaj & Fazliu, 2021).

This article is structured through following sections. After the brief introduction and problem statement, Section 2 provides a background to the inquiry of this research question through an overview of the existing literature specific to architectural education which outlines its current state and nuances from various realms of the field. Section 3 presents the methodological approach adopted for this study and the rationale behind its choice. Section 4 provides the results of the findings through a discussion of eight broad attributes of CBE from other fields of its current practices which can be related to the inherent requirements of architectural education. Section 5 concludes as a discussion of challenges, criticism and implications. A summary of suggestions is presented along with a note on future research.

## Literature review

With an aim to impart quality higher education, the National Education Policy 2020 echoes the objectives of UNESO and strongly advocates the need to develop good, thoughtful, well-rounded, and creative individuals ( MHRD, 2020). The making of a professional like an architect, incrementally begins from a school education. As an acknowledgement of the same, the NEP 2020 envisages a significant shift in the educational ecosystem in India at all levels, where the focus has now shifted from *‘content’* to *‘competency’.* It lays emphasis on a competency-based education right from the school environment and mandates to prepare students for the 21st century skills. It highlights the need for an assessment system that encourages real learning for real life situations, where assessment becomes the driver and motivator of change in teaching quality in the classroom. This systemic shift in perspective must make a way towards higher education, and bridge that gap for architectural education as well.

### Architectural education in India

As J. Mehta (2006) explains, Architectural knowledge and practice in India has been traditionally imparted and transferred through earlier generations in an informal manner, through apprenticeship and a master-pupil relationship. Before the beginning of a formal system of architectural education, the traditional and vernacular systems have prevailed. For the purpose of training artists and draftsman in reproduction of European classical arts, institutes like J.J. College of Architecture were opened in 1913, which introduced the initial model of formal and structured curriculum. The formation of institutions like Indian Institute of Technology, Kharagpur introduced a technological and scientific spirit. With such directions the utilitarian and problem-solving approaches began taking over some of the uniqueness of the profession which is a fusion of humanities, aesthetics and liberal arts ( Mehta, 2006). While different schools of thoughts have indeed made various kinds of qualitative contributions towards the evolution of architectural education in India, it has largely remained a reduced extraction of its regulating frameworks mostly dominated by quantitative and technical aspects.

Architectural Education in India is governed by the Council of Architecture, a statutory body of the Government of India, through guidelines prescribed by ‘Minimum Standards of Architectural Education’. Ar. Habeeb Khan (2019) in his article, *‘Nemesis in the genesis: Reforming architectural education in India’* highlights how the Minimum Standards of Architectural Educational is effective in assessing the quantitative aspects, but for the lack of attention to qualitative aspects the education is suffering ( Khan & Khan, 2019). While the standards allow ample room for institutions to develop their own curricular content as per their pedagogical ideologies, barring some of the notable educational practices most of the institutions revolve around adherence to the *‘minimum’* of broadly defined aspects.

Studio and Theory are the two prime categories of coursework in architectural education. Institutions try to adopt their own methods of delivering content as per the opportunities and challenges pertaining to organization, resources, and above all, the nature of vision they keep as providers of education and its experience. The assimilation of acquired skills and knowledge is tested in the industry, where practitioners often voice a concern for the quality of graduates coming out of the educational domain ( Chandavarkar, 2018). Apart from quantitative measures of performance, there aren’t any specific standard qualitative parameters which define or validate the quality of a learner’s progression. The conscious focus on the word ‘competent’, an explicit expectation of the Charter for Architectural Education (UIA), seems to be missing.

Explicit development of qualitative aspects and an intentional emphasis on the affective domain is very important in acquiring abilities for a learner’s progression. There is a need for a focused approach towards planning and an intent towards execution of a coordinated teaching learning framework, through its curriculum and pedagogy, which explicitly aims towards identification and assessment of such competences, which can enable its development. The study of current state architectural education in India points towards gaps in how architects are educated towards acquiring the qualitative dimensions of learning, in the definition of quality in learner progression, in mechanisms for building students’ competencies effectively during their studies, and in the assessment of such abilities.

### Current scenario in architectural practice

Like any other professional field, the role of knowledge integration in architecture is very important. As described by A. Salama (2008a) the production of architecture takes place in the field of tension between reasoning, feeling, and instincts, as integral modes of being. The holistic idea of ‘building’ (i.e. a ‘verb’) emanates from the conceptualization, coordination and execution of necessary abilities, while the act of building is rooted in humane tradition. Hence, the pedagogy for architecture must be focused towards manifestation of such integrations ( Salama, 2008b). At the 2019 Pune (India) conclave of concerned practitioners, educationists, young professionals and others working in allied fields, gathered to contemplate how architecture today is disconnected from its ethical foundation of being the profession that accepts responsibility for the stewardship of built environment, and is mired in a self-absorbed pursuit of personal glory and material acquisition instead of offering widespread and equitable value to nature and culture ( Conclave, 2021). Such voices reflect the state of values as perceived and practiced by the professionals and learners alike.

Ar. Prem Chandavarkar also points out, how we need to have a different mind-set to tackle the challenging demands of today’s rapidly changing environment and the need to produce professionally trained architects and designers ( Chandavarkar, 2018). Being a professional field, the need to effectively demonstrate the acquired knowledge, skills, and attributes is extremely critical for a holistic sustenance of the built environment. Such training and response mechanism from the architects will require a conscious awareness of their strengths and weaknesses as individuals with certain capabilities. While exposure to obtaining such attributes must be inculcated beginning from the educational journey, their conception must be rooted in the awareness of the broader expectations from the profession. So, education and practice share a symbiotic relationship, as well as responsibility, where one of the prime focus for these two spheres has to be around the definition and development of the set of competencies required by an individual to make meaningful contributions to our built environment.

### Society and architectural education

Viewed from outside of the academy as a social institution, education in post-industrial society is student centred, technologically enhanced, outcome driven, best-practice oriented, evidence based, and assessment validated. Hence measurable program learning outcomes become fundamental to the post-industrial/postmodern curriculum. Citing assessment as an ongoing activity which should aim at enhancement of the discipline, J. Mehta (2006) emphasizes on the need to create meaningful learning experiences and the role of systems based on competence as effective means to establish program coherence. Practices based on critical inquiry, self-discovery through reasoning and building empirical evidences of how learning is accomplished can also help institutions in mitigating the mounting pressure from accrediting agencies, government funding, employers and public sector, for accountability and transparency in educational systems which will eventually benefit the society as a whole ( Mehta, 2006).

### Role of curriculum in making of an individual

As pointed out by J. Mehta (2006), architecture is not merely a technical activity, but a value loaded activity, as it responds to the human needs and imaginations. Hence, the education of an architect and the curriculum it follows, must be much beyond the needs of the profession. It should not only provide for the basic competencies required by the discipline, it has an obligation to foster a culture of healthy criticism and ideation ( Mehta, 2006). Ar. Prem Chandavarkar (2018), refers to skills as “a necessary but not sufficient condition”, stressing on the need to go beyond skills and explore the value-based propositions which enable critical thinking, to build attitudes and temperament for life-long learning ( Chandavarkar, 2018; CSE, 2020). Hence, the need to address such gaps point towards a shift in the way education of an architect is conceptualized. It brings in focus the questions around the curriculum, and its integral structures which can facilitate the provision of such kinds of learning. To tackle such concerns, CBE has been widely used across the globe in different forms ( Ford, 2014). Although the concept isn’t entirely new, the lack of one standard way of conducting it, presents a challenges of reference points, but also provides a unique opportunity to assimilate best practices for formulating educational structures which are focused towards conscious development of relevant competencies.

## Methods

The objective of this article is to explore integral aspects of a CBE from other disciplines to understand their potential relevance with architectural education in India and identify attributes of CBE, as an outcome, which can be relevant for architectural education in India. This narrative review approach follows a qualitative data analysis process, as described by Ritchie et al ( Ritchie, Lewis, McNaughton, & Ormston, 2014). The following study design, data collection and data analysis method has been used to develop an understanding towards our research question.

### Study design

Owing to its limited or lack of adoption in field of architecture, there is a need to synthesize various aspects of CBE and integrate relatable experiences from various contexts and disciplines which have adopted it. Since such a practice is often domain-specific, such synthesis will require a nuanced understanding and description of qualitative interpretation of under-researched topics which can offer new insights and ways of thinking from robustly researched fields. In recognition of this need, a qualitative grounded theory approach ( Charmaz, 2009) and a non-systematic narrative review methodology was chosen for gathering and synthesizing a broad range of complex information to develop an overview, which can pave path towards focused areas of further systematic inquiries and future research questions about important aspects of adopting competency-based systems. The flexibility and rigor of a narrative review provides perspectives on exploring new insights for under-researched fields. It allows assimilation of various elements of data to establish coherent explanations for further research.

The initial search started on 9 ^th^ July 2022 using different keyword searches on multiple databases like Scopus, Google Scholar, ERIC, ResearchGate, Academia, Wiley Online Library. Considering the purpose of the study conference proceedings were also referred to gather a wide range of view from available research.

### Inclusion-exclusion criteria

The study included relevant literature which documented various fundamental aspects of CBE from the stand point of its conception, transition and execution. Since most of the documents broadly outlined more than one aspect the following concepts were derived as themes for the research question for making decisions around study selection; definition, origins, evolution, important milestones, characteristics, approaches and implications on teaching-learning, existing frameworks and models of assessment, challenges and criticism. Only full text documents were included for analysis.

The search result extended to included findings from a range of multiple disciplines like Medical education, Engineering, Social Science, Vocational Training, etc. from different regions and countries like Europe, USA, Russia, United Kingdom, Australia and Korea where specific organizations and regulatory bodies have focused their works towards the defining specific competencies for their respective regions with a suitable supporting framework. Keeping in mind the evolutionary phases of competency-based systems, type of sources included all type of research documents such as journal articles, conference proceedings, essay, technical report and guides, published in English language between 2000 and 2024. Even though it is not mandatory for this non-systematic form of narrative approach, we have broadly tried to follow the PRISMA 2020 checklist to adhere to the effective measures of reporting and guide the research for avoiding bias as far as possible. Since the study does not intend to critically review the identified literature, we have not followed all steps of the PRISMA 2020 checklist.

### Data collection

The documents were collected in independent online searches towards the following five categories; Architectural Education and Pedagogy, History and Disciplines of CBE Overview of CBE, Frameworks and Assessment in CBE, Transition from traditional education to CBE. Articles were manually screened and shortlisted after reading full texts and sorting the findings in these five categories ( Table 1). The selection of manuscripts for the review was to capture the broad aspects of CBE, instead of critical analysis of identified literature.

**Table 1.** Categories of search.

Categories of search		Screened	Shortlisted
1	Architectural Education and Pedagogy	38	2
2	History of CBE across Disciplines	50	37
3	About CBE	62	11
4	Framework and Assessment in CBE	41	9
5	From Traditional Education to CBE	86	17
		277	76

### Data analysis

Complete document files were independently studied by the three researchers. For appraisal of the included studies and to avoid the risk of bias, the Scale of the Assessment for Narrative Review Articles (SANRA) tool was used, which helped towards identification of the final full text included in the review ( Baethge, Goldbeck-Wood, & Mertens, 2019). Content was thematically analysed in the five categories (mentioned above) to address the purpose of this study. SANRA is an open access tool and the document explaining its instructions can be found at: https://www.aerzteblatt.de/down.asp?id=22862.

The PRISMA 2020 checklist and flow diagram has been used to report the findings of this study ( PRISMA, 2020) ( https://www.equator-network.org/reporting-guidelines/).

## Results

**Figure 1.**
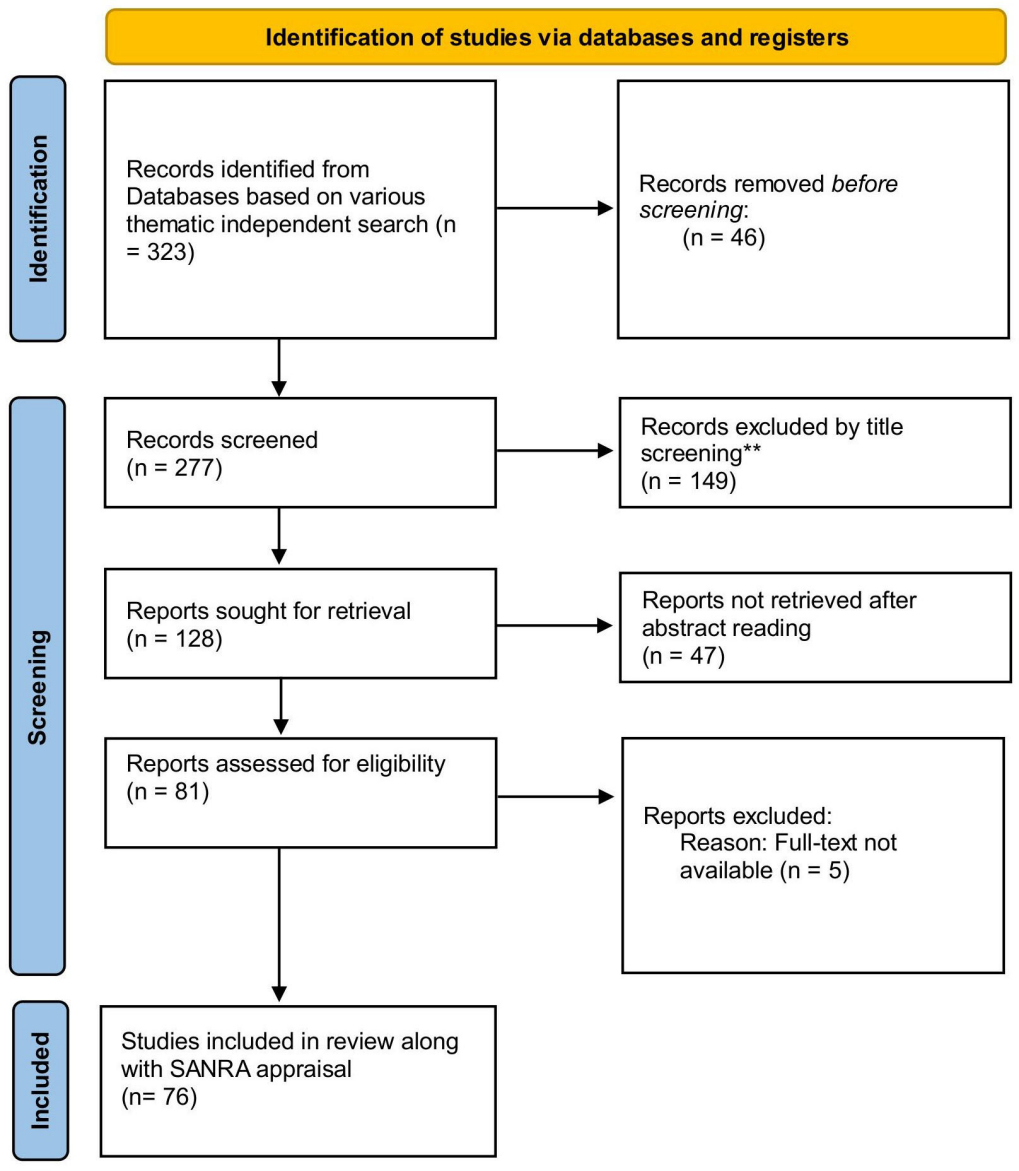
PRISMA 2020 flowchart showing results of the search.

The initial independent searches yielded a total of 323 documents in the 5 categories, i.e. Architectural Education and Pedagogy (n=56), History and Disciplines of CBE (n=51), Overview of CBE (n=62), Frameworks and Assessment in CBE (n=66), Transition from traditional education to CBE (n=88). After title screening 46 documents were excluded and a total of 277 document were retained for further screening. After screening of abstracts, 137 documents were excluded, and 140 documents were sought for full text reading. After screening through full reading stage, 76 documents were found eligible for the purpose, and were included in this narrative review. i.e. Architectural Education and Pedagogy (n=02), History and Disciplines of CBE (n=37), Overview of CBE (n=11), Frameworks and Assessment in CBE (n=09), Transition from traditional education to CBE (n=17). The results are presented below in a flowchart following the PRISMA 2020 template ( Figure 1), and a summary of the included studies has been presented in Annexure 1.

The included literature finds its origin in different disciplines and regions between a timeline of 2000 to 2024, with a wide range of purpose in the focus of its documentation. The type of documents included are Articles (n=59), Book (n=1), Case Study Document (n=1), Reports (n=7), Essay (n=1), Reference Documents (n=5), University update (n=1), call for paper (n=1). Most of the information comes from the field of Medicine (n=30) and the least from vocational fields (n=3). We could find a few relevant documents from the discipline of Architecture (n=4). Geographically, majority of the included literature comes from USA (n=39), India (n=8) Canada (n=7), while others combine from Russia, Canada, Netherlands, Australia & New Zealand. While the CBE has been in contention earlier than 2000 as well, there has been a surge in the perception of its relevance since 2010. Of the identified literature, 10% comes from a timeframe between 2000-2010, while the remaining 90% is spread out between (2010-2024), with a consistent and continuing rise in such publications.

Through a broader understanding of the several critical parameters discussed in the literature collected in 5 categories, we could observe a presence of 11 different themes ( Table 2) across these documents, like; (a) Discussion of key concepts (n=39), (b) Definitions (n=21), (c) History or Origins (n=16), (d) Regulatory environment (n=11), (e) Challenges and Gaps (n=11), (f) Characteristics and Principles (n=30), (g) Transition points (n=9), (h) Implications of Teaching and learning (n=19), (i) Assessment frameworks and models (n=25), (j) Future directions (n=23), and (k) Criticism on CBE (n=8).

**Table 2.** Themes from the literature.

Themes from the literature	
1. Discussion of key concepts	**39**
2. Definitions	**21**
3. History or Origins	**16**
4. Regulatory Environments	**11**
5. Challenges and Gaps	**11**
6. Characteristics and Principles	**30**
7. Transition Points	**9**
8. Implications on Teaching and Learning	**19**
9. Assessment Frameworks & Models	**25**
10. Future Directions	**23**
11. Criticism	**8**

## Discussion

The findings have been synthesised and presented here as a narrative in 9 themes which reflects the overview on the current state of knowledge of CBE with respect to its; (1) Definitions from different contexts, (2) History: origins and transition from traditional to CBE, (3) Transitions, (4) Regulatory environments, (5) Characteristics, (6) Approach and implications on teaching and learning, (7) Assessment frameworks and models, (8) Challenges and Criticism, and (9) Implications. These segments aim to capture some of the key ontological and epistemological concepts which can `provide the necessary rationale leading towards the understanding of CBE, and its potential relevance to architectural education in India.

### Definitions

A *“skill”* is about doing something well, *“Knowledge”* is the known information, and an *“attribute”* is an inherent quality and is often expressed through what you think, do and feel. Together, these three elements make up a competency. Competencies are expressed in ways that can be observed, measured, associated with different real-life environments, transferred and are performance-based. To understand competence/competency, from an educational perspective it is equally important to inquire upon the notions of the word curriculum. It derives its root in the verb ‘ *currere’* in Latin. The meaning of the word involves connotations of ‘movement’ and ‘journeys’. R R Burns, in his article, explores the entomological roots which explains a deeper understanding of the word curriculum. Curriculum can also be related as a journey to the places one may not think are important, but gathers significant experiences as a result of its dynamic socio-temporal nature which is embodied in the time, space and the manner in which that time is spent ( Burns, 2020). Spady (1977) in his article, summarizes the approach called ‘competency-based education’ where he emphasizes on the need to build suitable mechanisms to facilitate appropriate diagnosis of weaknesses and remediation.

Defining ‘competency’ and an ‘educational competency’ is a complex challenge. As per PISA (Program for International Student Assessment), it is more than knowledge or skill. It is more about developing abilities to meet complex demands. Irina A Kedrova, in her research, explains a Russian perspective through the words of A.V. Khutorsky (2004) who understand competency as an individual’s preparedness to apply the knowledge, skills and external resources for enabling effectual actions in certain life situations ( Kedrova, 2016). In United States of America, CBE is perceived in terms of having focus on learning outcomes, instead of time spent in a classroom ( Education, 2015). Competency is concept related to a person, while competence is a concept related to work ( Armstrong, 2005). The definition of competencies can vary as per interpretation as it needs to assess critical human characteristics as *“quality”, “character”.* Competence is a holistic concept which reflects an individual’s capacity and abilities to perform effectively ( Ten Cate & Scheele, 2007; Ford and Meyer, 2015). A model of professional competence can be categorized into five types; (a) Cognitive, (b) Functional, (c) Personal, (d) Ethical, and (e) Meta-competence. In contrast to the work done by Russian and Kazakh scientists, other scholars have focused on the practical orientation of this issue ( Makulova, et al., 2015).

Architectural practice embodies process, organization, business, conviction. Apart from focusing on the products of the practice, Architects engage with the contradictions between responsibilities of ethical nature and bearing accountability towards the stakeholders, the challenges of the practice and intricacies of architectural imagination, the aesthetical and imaginative foundations of the field makes it incumbent upon the architects to possess ecumenical proficiencies and knowledge, which largely points towards the broader expectations of being ‘competent’. In its most basic form, competence is the quality of being competent, while competition is the act of competing. The word competency also derives its roots from the Latin word *competentia,* which means “meeting together, in agreement and symmetry” ( Seng, 2021).

### Origins

Outcome Based Education (OBE) which defines learning in terms of outcomes can be considered as the precursor to CBE. Beginning with its roots in behaviourism, different authors have mapped the evolution of CBE in terms of its societal and theoretical origins. The development of competency-based approaches can be understood outside of the realm of education and training through practices like craft guilds, apprenticeship, etc., where criteria for competence and performance have been defined and developed for certain vocations and positions ( Nodine, 2016). As a society, even as the idea of competency has existed since over a century, according to Houston (1974) CBE has evolved as a cultural trend towards ‘accountability’ and ‘personalization’ in American societies. As a political catalyst, a range of commentators also credit the development of CBE to the effects of the launch of Sputnik on the American imagination, which triggered an educational reform in the America. The passing of National Défense Education Act in 1958 has been seen as the first formal step which emphasized on mastery in education and training. Another stimulus was the enactment of the Vocational Education Act in 1963. It triggered development of educational models which were characterized by ‘the precise specifications of competencies’. The Elementary Teacher Education Models (1965) a program of the United States Office of Education (USOE) formulated the Performance-based Teacher Education (PBTE) movement ( Hodge, 2007).

Theoretically, the fields of behavioural psychology and systems theory have important influences on the origins and development of CBE. As mentioned by Hodge (2007), most of the psychologists who worked towards the problems of efficient training in the United States military had a behavioural premise. They worked with the rationale that our knowledge is ultimately built on our sensory experience. In 1913 the American scientist John B. Watson aimed to study human psychology strictly in terms of observable behaviour. Descartes’ theory, identifies the necessary connection (reflex) which exists between a given external influence (stimulus) and the resulting reaction (response). Contributions by Thorndike (1911), Pavlov (1927), Skinner (1953) in terms of theories on behaviourism have positively influenced the general approach of CBT. Bertalanffy (1968), Gagné (1962) and Crawford (1962) through their philosophies on Systems Theory emphasized on the inter-disciplinary amalgamation ( Hodge, 2007). Traditionally, for the eventual purpose of application, the studio-based learning philosophies and the hands-on approach has dominated the knowledge generation and integration for learners in the field of architecture. The interdisciplinary nature of architectural practices, has always drawn attention towards the learnings of cause-and-effect principles and interpretations from different fields to expand its horizons which have a far wider impact on human condition. Since the intention of CBE is focused towards development of individual characteristics for betterment of outcomes, a lot can be learnt from such practices in the future course of its evolution.

### Transitions

The programs following a CBE approach in American higher education can be classified in following three phases: (a) Teacher Education programs (1960s onwards); (b) Vocational Training and Education programs (1970s onwards); and (c) more recent “Direct Assessment” programs as Online or Hybrid models, which have been propelled by the advances in adaptive learning technology. Programs offered by Western Governors University and other such institutions, can be seen as part of the latest phase in the development ( Nodine, 2016). In its transition CBE has followed different approaches in different regions, where the key differences have emerged in terms of the ‘form of education’ and its ‘content’ ( King, 2021). The approaches adopted in USA have typically meant flexible, personalized, self-paced, skill-based education. Whereas the other approach spread across Europe and Russia are associated with the Bologna System of Higher Education, which comes from the Bologna Process ( Bernikova, 2017).

Transitioning from a traditional studio and apprenticeship-based practices and in view of the regulatory environment, the scale of architectural education in India has encouraged several institutions to turn to Outcome-based Education (OBE) model. While keeping its focus on the outcomes, CBE redefines and prioritizes the nature of learnings in terms of acquisition of competencies as the indicator of the outcome, which are not just intended to be more observable, but also measurable so as to enable the adoption of tangible practices towards development of such attributes which promote life-long learning.

### Regulatory environments

It is important that the regulatory bodies develop supportive regulatory formats which meet the needs of competency-based programs ( Cunningham, et al., 2016). As explained by Nodine (2016), in the US higher education ten institutions were chosen to initiate CBE in 1968 to provide training programs for elementary school teachers. These pilot programs, which also represents the initial competency-based models for higher education, aimed towards improving quality of aspects related to teachers. In 2013 the Experimental Sites Initiative (ESI) program, issued a reference manual on CBE to make using federal student aid for such programs easier ( Nodine, 2016).

CBE began to gain more prominence with the development of Western Governors University (WGU) in 1995. Alverno College (1970s), Thomas Edison State (1980s), Charter Oak State College (1990s) and Southern New Hampshire University’s College (2000s) are some of the other programs known CBE programs. As Sasha Thackaberry highlights, how the Higher Education Act in 2005 made it possible for “Direct Assessment” programs. Southern New Hampshire University (USA) was the first college approved with this option in 2013 ( Thackaberry, 2016a, 2016b). Several voices in India call upon the need to build on the qualitative aspects of architectural pedagogies and assessment of learning. The instances from other fields and countries highlight the critical role played by the regulatory environments in this regard, which can turn out to be the catalyst for change. The need to recognize the far-reaching benefits and consequences of programmatic assessment are paramount. NEP2020 opens up new avenues for the same.

### Characteristics

As explained by McGaghie, an educational program’s goal is competence, and a curriculum provides the means by which competence is to be attained ( McGaghie, et al., 1978). A competency-based approach is focused towards principles of personalized and mastery learning, where the development and characterization of competencies is expected to be career relevant, professionally broad and specific to an attribute ( Cunningham, et al., 2016). Several authors have elaborated the various descriptions and characteristics of competencies and CBE. With reference to the medical field, Olle Ten Carte and Stephen Billett explain how context facilitates and often determines the translation of knowledge into its enactment. Competencies can be looked at as the will to use the canonical knowledge in situational environment ( ten Cate & Billett, 2014). Patricia A. Book (2014) in her report *“All Hands on Deck: Ten lessons from early adopters of competency-based education”* captures snapshots of the experiences from seven competency-based post-secondary programs and reflect from their exemplary work ( Book, 2014). Malulova et al. explains how the components of the “four pillars of education” by Jacques Delors serve as the pre-requisites for competency and competency-based approaches ( Makulova, et al., 2015).

Haris Saud and Ruth Chen (2018) in their integrative literature review cite observations by Lawrence Stenhouse who argued that it is not possible to develop values, understanding, and judgement solely through behavioural goals. Even if assessment of the affective attributes can’t always be objectively performed, they are equally important ( Saud & Chen, 2018). Such characteristics and attributes are integral to the objectives and expectations from learners of architectural education. With the classification and categorization of the nature of competencies, the overall aim of developing individual capabilities in a holistic manner remains the ultimate goal of CBE as well. Relevant criteria for learner performance and methods of assessment can be developed through appropriate methodologies for architectural education in India to enhance the awareness towards the necessary means and approaches for attaining it.

### Approach and implications on teaching and learning

In CBE the teacher is supposed to adopt a coaching role who guides the learning processes for a student. The responsibility of learning belongs to the learner, while it must be facilitated by the instructor. This transition requires a paradigm shift in perceptions and attitudes from both stakeholders ( Clarke, 1990; Biemans, et al., 2004). As the initiators of this approach, the value of faculty willingness and training is an integral aspect in the course of development of CBE. Nadia Serdenciuc has enlisted a range of abilities that would be relevant as a possible teacher’s profile from a CBE perspective ( Serdenciuc, 2013). Steven Hodge (2007) in his article cities Kornhauser (1922) who made a number of recommendations to reform apprentice training while educational theorist Carroll (1963) provided the first complete model of mastery learning. Realizing its potential, Bloom (1956) built on this model by identifying five factors which each mastery strategy must deal with; (1) Aptitude, (2) instructional quality, (3) How one understands instruction, (4) Persistence, and (5) Learning time. Bloom’s advocacy of mastery learning represents a humanist contribution to CBT ( Hodge, 2007).

**Figure 2.**
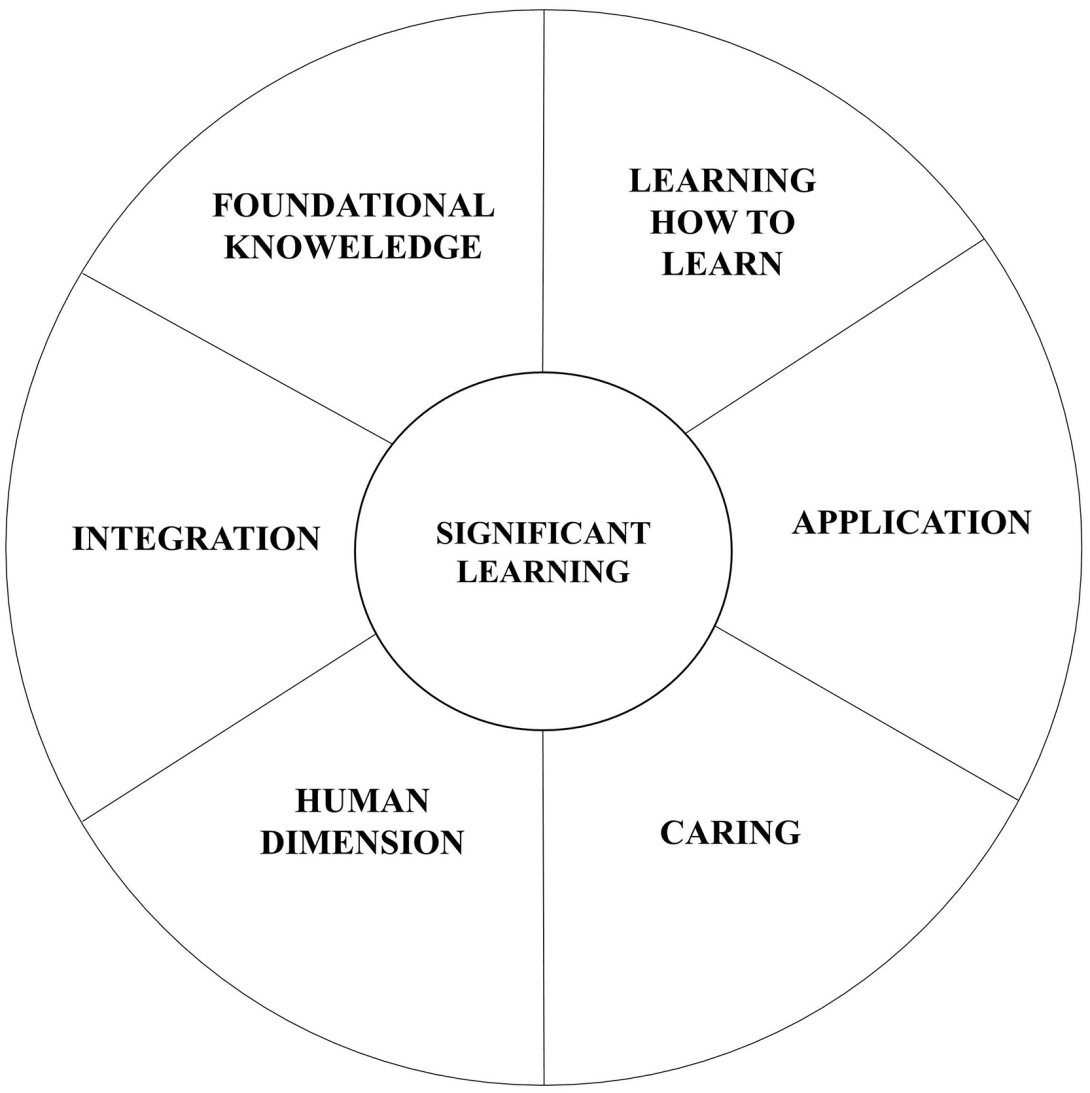
Six Domains of Fink's Taxonomy of Significant Learning.

Kate Ford in her technical report has identified the Degree Qualifications Profile (DQP) by Lumina Foundation’s as one of the important learning frameworks which outlines educational outcomes in terms of what graduates should know and can do. The DQP focuses on specific learning areas to organize the competencies of a program ( Ford, 2014). For a learning to reach a specific level of performance it is important to know and map how one can get there. Lockyer et al. (2017) have discussed the importance of Miller’s (1990) contribution who related the four levels of learning as a represented in the form of a pyramid. Beginning with what the learner “knows,” the hierarchy then proceeds through levels of “knows how” and “shows how” before reaching the highest level, “does” ( Lockyer, et al., 2017). Jeremy Branzetti et al. (2019) attempt to focus beyond the notions of competency and its assessment, saying it is equally important to adopt a focused and a meaningful approach towards curriculum design. According to him, curriculum can be a mitigator to assessment. Fink’s model of taxonomy and its six domains, as learning areas, could be an effective way to achieve this outcome instead of the widely used Bloom’s taxonomy ( Branzetti, et al., 2019). Figure 2 explains the 6 domains of fink’s taxonomy of significant learning. These domains can be understood for its relevance with architectural education as well.

### Assessment frameworks and models

As Wiggins (1998) says, the aim of assessment should be to improve performance of the student instead of merely auditing it ( Wiggins, 1998). The relevance of content-referenced measures of performance assessment in CBE has been strongly explained and established by various educators and scholars. Glaser (1962) has proposed that the testing should indicate whether the students have developed the ‘behavior’ expected or intended in the design of the coursework ( Glaser, 1962). Citing a case of the medical field, Norcini (2005) explains how higher order assessment would require direct observation, structured feedback on performance or skill-based evaluation is simulated on real patients ( Norcini, 2005; Norcini & Burch, 2007; Cate and Regher, 2018). Dilmore et al. (2013) in their article describe the CBE Framework Development for a Clinical and Translational Science Institute (CTSI). As a certificate and degree-granting program, they followed the incorporation of the following three steps in their structure; (1) Curriculum Development, (2) Curriculum Alignment, and (3) Curriculum Support. Lomisa et al. (2017) recommend the framework of ‘Milestones’ which are “criterion-referenced” set of performance benchmarks.

**Figure 3.**
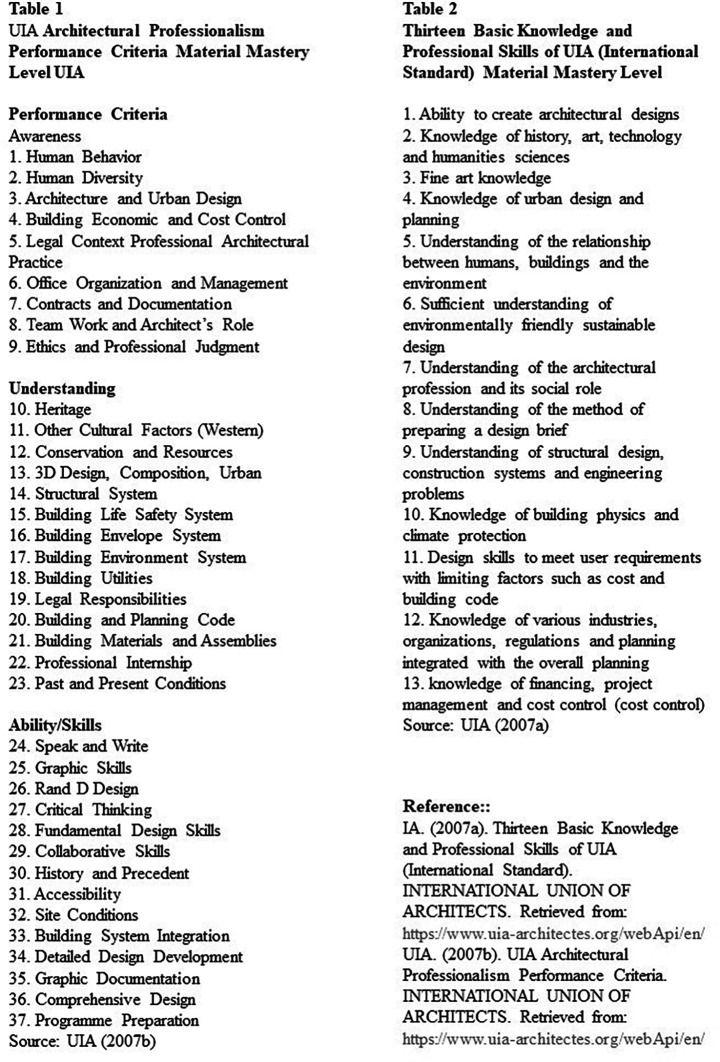
UIA criteria. Source: UIA. (2007a). Thirteen Basic Knowledge and Professional Skills of UIA (International Standard). International Union of Architects. Retrieved from: https://www.uia-architectes.org/webApi/en/. UIA. (2007b). UIA Architectural Professionalism Performance Criteria. International Union of Architects. Retrieved from: https://www.uia-architectes.org/webApi/en/

These can be applied to various programs, courses and settings. It can improve the process of trend identification and allow stronger evidence to be generated which can in turn prompt the student to act ( Lomisa, et al., 2017). The Entrustable Professional Activities (EPA) Framework, developed by Olle ten Cate for programs in the medical field, is becoming a widely accepted framework. It leads to recognizable outcomes which are expected by the community from the competent professionals. It includes a list of competencies and in totality they encompass a given domain ( Ten Cate & Scheele, 2007; Gruppen, et al., 2016). Dwijendra, in his research article which captures aspects of learning around architectural education in Indonesia, cites the case of UIA, where high-performance standards of professionalism are used by UIA to describe an architect’s professional capabilities. Figure 3 shows these requirements which include 13 fundamental professional knowledge and abilities of UIA international standards, 3 mastery levels, and 37 items ( Dwijendra, 2021).

**Figure 4.**
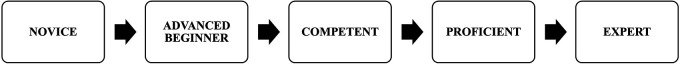
The Dreyfus model of skill acquisition.

Many institutions and alternative educational providers are exploring the intricacies of developing a CBE programs. An article by A. Sasha Thackaberry outlines how the increasing sophistication of CBE programs has largely been possible due to technological improvements. ( Thackaberry, 2016a, 2016b) The ACGME refers to the Dreyfus Model (1986) to outline competencies for its programs which require guidelines on methodology training. Hubert and Stuart Dreyfus, who were assigned by the U.S. Air Force, came up with the concept to explain how a pilot’s knowledge and abilities grow. This model, has five levels of proficiency ( Figure 4), in an increasing order of mastery learning which can be referred as milestones of a learner’s progression ( Sultan, et al., 2019).

### Challenges and criticism

For implementing a mastery approach educational institution need to clearly communicate the expectations of mastery and levels of achievement and identifying ways in which students can demonstrate such proficiency or mastery ( Nodine, 2016). All competency-based programs are different and hence it poses a challenge to accreditors. From a secondary school education perspective, teachers have felt that competency-based assessments forces them into endless cycles of assessment and that deciding the levels of achievements as well as the design of assessment instrument have been difficult tasks. This also points towards the challenges associated with building a pool of skilled and dedicated faculty which can train and assess the level of competency in a trainee ( Clotilda, 2021; Pitman, et al., 2000; Oroszi, 2020).

Based on the observations from the current literature available, Hoang and Lau (2018) have identified “Reductionism” and “loss of authenticity” as critical challenges for developing the curricular structures and corresponding assessment tools ( Hoang & Lau, 2018). The literature on CBE points towards a different kind of engagement by faculty, most importantly in the domain of willingness. Ford highlights how the described changes in traditional faculty roles, have resulted in the faculty themselves being some of the chief critics of CBE ( Ford, 2014). In a literature review of documents published between 1966 to 2002, Carraccio et al. (2002) observed that application of competency-based models to medical education often end up being stalled at the conceptual level ( Carraccio, et al., 2002).

Ernest L. Boyer, then-president of the Carnegie Foundation for the Advancement of Teaching, during his lecture in 1993, urged educational systems to move beyond the old Carnegie units as the learners still fail to acquire a more encompassing view of knowledge and life. As Cunningham et al. (2016) emphasizes, the development of CBE programs must focus upon formulating process towards practicing alignment, strengthening validity, defining faculty roles and supporting students through the assessments ( Cunningham, et al., 2016). Citing the Cleveland Clinic Lerner College of Medicine, who decided to change the system of grading medical students, focused on developing appropriate performance standards, Olle ten Cate roots for the use of “narrative description” with relevant behavioural milestone descriptions, which can be converted into a 9-point scale ( ten Cate & Billett, 2014). Citing Hattie and Timperley (2007), Peter Bohain (2018) highlights the importance of ‘feedback’ on assessment tasks, and a need to reduce the emphasis on grades or marks as they contain little information about improvements that can be brought about for reaching the learning goals ( Bohain, 2018).

Competency-based evaluation can make a significant contribution to finding, choosing, and developing brilliant people ( van der Merwe, 2002). Based on how candidates actually perform in their allocated job positions, the assessors judge whether candidates meet the predetermined competency standards during the assessment process ( Gonczi, 1994). Conducting a good competency-based evaluation, however, is difficult ( Suhairom, et al., 2014). First of all, not all competencies can be seen in plain sight. Second, certain commercial organisations or professions have very broad and diverse job responsibilities. Making sound judgements has become challenging as a result. In order to precisely and successfully estimate an individual’s competency levels in carrying out particular jobs and duties, evaluation methods as well as the assessors, must be thoughtful and dynamic ( Baartman, et al., 2006; Clary et. al, 2011). Additionally, sufficient evidence of performance, both in terms of quality and quantity, must be gathered in order to make accurate assessments ( Gonczi, 1994; Lee, 2018). The assessment and improvement of competencies is a continuous process. For the purposes of competency assessment, the created competency information must be properly arranged and presented. A competency typically has three parts: a definition of the competency title, an explanation of the visible behaviours that can be utilized to show competency mastery, and a thorough discussion of the skill’s performance levels ( Campion, et al., 2011). The process of defining competencies is a rigorous process which requires awareness and complete involvement of the faculties, since they need to be aligned with the finer aspects of learner progression.

With an increasing number of educational environments adopting CBE, several integral aspects of this framework are being reported as challenges to deal with. For enhanced accountability and justifying its relevance, CBE is focused towards making education less time-bound, and primarily driven by development of learner competencies ( Hoffman, Dedow, & Boscamp, 2024). For systems which are currently oriented towards time-based progressions this can be a major visionary challenge. Additionally, such systems also need to acknowledge the role of incremental development in learning for improving the educational outcomes. The effectiveness of such systems requires removal of potential biases in judgments through well-thought out assessment framework ( Holmboe, Osman, Murphy, & Kogan, 2023).

CBE environment emphasizes involvement of all stakeholders and a transformative approach towards hidden curriculum for decision-making on various facets of formulation and implementation ( Dandekar, Mahdi, & Chacko, 2023). The willingness and preparation of faculty towards overcoming the familiarity of the current systems is crucial for the transition towards CBE. Positive reflection and readiness for implementation can be challenge for the faculty who are the critical facilitators of CBE as their commitment will immensely impact the overall outcome ( Alkandari, 2022; Stoffman, 2022). Training quality, for students as well faculty, can also significantly add to the outcome of such transitions. Hence pedagogical interventions need a conscious consideration ( Cao, Vu, Nguyen, & Nguyen, 2022; Paquet, et al., 2023).

While CBE itself is a systemic change in perception and organization of educational environment, it must be viewed as a facilitator to prepare professionals for leading the change in our complex and dynamic environment which continually demands challenging transformations ( Dagnone, Meschino, Rooyen, & Scheele, 2020). CBE is often criticized for its resource intensive requirements and lack of resource is often reported as a serious challenge. While such resources could be structural, staffing, financial, technological and environmental in nature, all educational environments are potentially different and they often require a context-specific approach. For long term sustainability, all potential barriers must be carefully explored before opting for and creating suitable CBE frameworks ( Ras, et al., 2023; Szulewski, Dagnone, & Schultz, 2023). Assessment is often seen as a burden and it requires a contemplative approach to implement and monitor. As a systemic change there is always a need for a strong leadership at the forefront of such transformations ( Stoffman, 2022).

### Implications

Several objectives from the Charter for Architectural Education by the UNESCO/UIA are coherent with the intention of the NEP 2020. The enhanced focus on imparting and strengthening the 21 ^st^ century skills in learners is one of the common objectives of these two policy-based documents which aim to foster global collaborations while adequately respecting the local. Architectural education is quite diverse in nature which not only involves an active multi-disciplinary engagement with best practices from across the globe, but it also mandates adoption of sensitive practices which have contextual implications. With a growing call for attention towards qualitative parameters, the mechanism for building student competencies during the educational phase becomes extremely important.

While such competencies may be exhibited and assessed more completely in the practice phases of the discipline, the mindset required for integration of knowledge and development of holistic values can be nurtured through a conscious awareness and judgement of one’s own strengths and weaknesses. Learner-centric educational systems require active involvement of approaches which can genuinely promote self-discovery and appreciate critical inquiry. The obligation of architectural education to holistically respond to various facets of human imagination and condition put an impetus of the nature of education one receives. Incorporation of CBE can provide a genuine response to foster this symbiotic relationship between education and practice to produce well rounded professionals.

### Suggestions

The objective of this article is to explore integral attributes of a CBE from other disciplines to understand their potential relevance to various challenges facing architectural education in India. Table 3 summarizes the various challenges of its context as aspects of the eight domains of this study, and attempts to provide a broad map of directions and outcomes as a suggestion which can be incorporated through the learning offered by the attributes of CBE.

**Table 3.** Summary of challenges, strategies and relevant attributes of CBE.

Challenges facing architectural education in India (Gaps)		Strategies (Directions)	Relevant attributes of CBE (Outcomes)
1	The conscious focus on the word competent. *(Definition)*	The will to use the canonical knowledge in situational environment.	*Levels of learning; Entrustable Professional Activities (EPA); Framework of ‘Milestones’ which are “criterion-referenced” set of performance benchmarks.*
2	Disconnect with the ethical foundation. *(Origin)*	A cultural trend towards 'accountability' and 'personalization'.	*A paradigm shift in perceptions and attitudes from both stakeholders; The value of faculty willingness and training.*
3	Lack of attention to qualitative aspects. *(Transitions)*	Assessment of the affective attributes.	*The testing should indicate whether the students have developed the ‘behaviour’ expected or intended in the design of the coursework.*
4	Individual awareness of their strengths and weaknesses. *(Regulatory Environment)*	Personalized and mastery learning.	*Direct observation; Structured feedback on performance; Skill-based evaluation*
5	The quality of graduates. *(Characteristics)*	Career relevant approach and inclusion of attributes which are professionally broad, as well as specific.	*Qualifications Profile; “Narrative description” with relevant behavioural milestone descriptions.*
6	Appropriate diagnosis of weaknesses and remediation. *(Approach and implications on teaching-Learning)*	Flexible, personalized, self-paced, skill-based education.	*Curriculum can also be related as a journey.*
7	Knowledge integration *(Frameworks and models of assessment)*	The translation of knowledge into its enactment.	*Formulating process towards practicing alignment, strengthening validity, defining faculty roles and supporting students through the assessments.*
8	Assessment *(Challenges)*	Evaluation methods, as well as the assessors, must be thoughtful and dynamic.	*The importance of ‘feedback’ on assessment tasks, and a need to reduce the emphasis on grades or marks; Encouraging expert judgment and understanding role of subjectivity.*

### Limitations

The research study focused on understanding broad aspects of competency-based education and its relevance to architectural education in India through a non-systematic form of narrative approach. Performing critical analysis of the identified articles was not the intention of this study, so we may have missed out on capturing some aspects of this study.

## Conclusion

Competency-based education (CBE), as a cultural trend towards accountability and personalization, is a systemic change and it requires a shift in perception and attitudes from all stakeholders. It can be a disruptive advance in higher education and the literature review points towards the following outcomes as strategies to be adopted. It requires programs to coordinates academic content and its delivery according to competencies, instead of conforming to conventional systems. CBE, at its core, envisions curriculum as a journey which can enable diagnosis of weakness and remediation, aspires to recognize and encourage better preparedness. With a prime objective of promoting mastery learning, CBE calls for coherent definitions of competency statements and development of qualification profiles for architectural education. Learner progression needs to be explicitly outlined though a system of milestone which embody the core characteristics of innovative teaching-learning models.

Strategies in terms of practices like ‘Competency Portfolio’ as “the transition to competency” is one of the key exemplars towards understanding different measures for developing a CBE environment. Expert judgment, which includes subjectivity, is unavoidable in discipline like architectural education, and it is a quality which can increases with experience. The validity of situation-specific activities and subjective judgements must find a place in the process of assessment, which has also been echoed by frameworks like Milestone development, levels of learning and Entrustable Professional Activities (EPA). Currently medical education in India has already begun to evolve its systems along the lines of CBE. Such measures demand experimentation, process orientation, dedicated involvement from educators, a radical rethinking in the manner of outlining our educational goals and intentions for developing the means to achieve them. The frameworks of assessment need to be dynamic and holistic in nature to provide a meaningful sense of feedback which promotes true lifelong learning and emphasizes development of an individual character.

### Future directions

Future direction could revolve around development of relevant criteria for learner performance and methods of assessment can be explored through appropriate methodologies for architectural education in India. CBE brings about structural changes in the educational environment which requires active involvement and coordinated participation from the entire team of instructors as a beginning towards knowledge integration and an attitudinal change in academicians is required to provide a better coordinated learning and assessment of affective attributes in terms of content, process and environment. Since the competency approach in education is still evolving, incorporation of such systems will mean a conscious and sensitive assimilation of the experience from different environments for a contextual application, along with the attributes of the NEP 2020 in India.

## Data Availability

The authors confirm that the data supporting the findings of this study are available within the article and its supplementary materials. This research article has followed a narrative review method and has adhered to the PRISMA 2020 reporting guidelines, as far as possible. The corresponding PRISMA checklist (word document), and flow diagram (word document), has been included as the underlying data. Figshare: Checklist for PRISMA_2020_checklist (RCBEAEI), https://doi.org/10.6084/m9.figshare.26023858.v2. The summary of articles included for study (Annexure 1). The SANRA appraisal data (Annexure 2) has been included as the supporting material in the form of extended data (word document and excel sheet). SANRA is an open access tool and the document explaining its instructions can be found at: https://www.aerzteblatt.de/down.asp?id=22862. These files can be found in the Figshare repository under the project titled ‘Relevance of competency-based education for architectural education in India’, with DOI: https://doi.org/10.6084/m9.figshare.26023858.v2 Licence: CC0.
