# Supplementary material for: Relevance of Competency Based Education for Architectural Education in India
Source: F1000Res. 2024 Nov 11;13:835. Originally published 2024 Jul 25. [Version 2] doi: 10.12688/f1000research.148617.2 (PMC11587234; doi:10.12688/f1000research.148617.2)
Supplement: Peer review history

# Reviewer report: Reviewer response for version 2

**DOI:** 10.5256/f1000research.174244.r339444

**Published:** 2024-11-22

**Authors:** Jayendira P Sankar

**Affiliations:** 1 Master of Business Administration, University of Technology Bahrain, College of Administrative and Financial Sciences, Salmabad, Bahrain

Yes, the review comments were amended.

Is the work clearly and accurately presented and does it cite the current literature?

Partly

If applicable, is the statistical analysis and its interpretation appropriate?

Partly

Are all the source data underlying the results available to ensure full reproducibility?

Partly

Is the study design appropriate and is the work technically sound?

Partly

Are the conclusions drawn adequately supported by the results?

Partly

Are sufficient details of methods and analysis provided to allow replication by others?

Partly

Reviewer Expertise:

The current research interests include Development Economics: economic growth, poverty, inequality, market failure, fiscal, economic and social condition, healthcare, education, employment sector, industrial and social infrastructure; Human Resource Management: employee welfare, work-family balance, consumer behaviour, retail marketing, corporate social responsibility, sustainability, and teaching-learning and assessments.

I confirm that I have read this submission and believe that I have an appropriate level of expertise to confirm that it is of an acceptable scientific standard.

# Reviewer report: Reviewer response for version 1

**DOI:** 10.5256/f1000research.162950.r307235

**Published:** 2024-10-24

**Authors:** Ngakan Ketut Acwin Dwijendra

**Affiliations:** 1 Udayana University, Bali, Indonesia

**Comments and Suggestions for Improvement**

1. **Literature Review:** The literature review is comprehensive but could be enhanced by including more recent studies on CBE, particularly those published after 2020. This would provide a more up-to-date perspective on the topic.
2. **Methodology:** The methodology section could be expanded to include a more detailed description of the inclusion and exclusion criteria for the reviewed documents. This would improve transparency and allow for easier replication by other researchers.
3. **Data Availability:** Ensure that all supplementary materials and data mentioned in the article are accessible to readers. This could involve providing direct links to the datasets or ensuring that they are available in an open-access repository.
4. **Citations:** Review and update citations to include more recent and relevant studies. This will strengthen the article’s foundation and provide a more current context for the discussion.
5. **Discussion and Implications:** The discussion could benefit from a more in-depth analysis of the potential challenges and limitations of implementing CBE in architectural education. Addressing these issues would provide a more balanced view and prepare educators for potential obstacles.

**Points to Address for Scientific Soundness**

- Clarify the criteria for document inclusion and exclusion in the narrative review.
- Ensure all supplementary materials are accessible and clearly referenced in the article.
- Update the literature review with more recent studies to reflect current trends and practices.

**Conclusion**

 The article makes a valuable contribution to the discussion on integrating CBE into architectural education in India. By addressing the points mentioned above, the authors can enhance the clarity, robustness, and impact of their work. Overall, the study is well-conceived and provides a strong case for the relevance of CBE in this context.

**Recommendation:** Partly

 This assessment acknowledges the article's strengths while highlighting areas for improvement to meet the highest standards of academic rigor and transparency.

Is the work clearly and accurately presented and does it cite the current literature?

Partly

If applicable, is the statistical analysis and its interpretation appropriate?

Not applicable

Are all the source data underlying the results available to ensure full reproducibility?

Partly

Is the study design appropriate and is the work technically sound?

Yes

Are the conclusions drawn adequately supported by the results?

Yes

Are sufficient details of methods and analysis provided to allow replication by others?

Partly

Reviewer Expertise:

Architecture, Education

I confirm that I have read this submission and believe that I have an appropriate level of expertise to confirm that it is of an acceptable scientific standard, however I have significant reservations, as outlined above.

# Response

**Published:** 2024-10-30

**Authors:** Nikhil Kohale

**Affiliations:** MSAP, Manipal School of Architecture and Planning, MAHE, Manipal, Karnataka, India

**IN RESPONSE TO SUGGESTIONS/ COMMENTS FROM REVIEWER 3 **(Mr. Ngakan Ketut Acwin Dwijendra, Indonesia)

 Dear reviewer,

 Thank you for your feedback and suggestions. Sharing our response to the revisions/updates we have introduced in the updated/revised version (v2). 

**Comment 1: **Literature Review: The literature review is comprehensive but could be enhanced by including more recent studies on CBE, particularly those published after 2020. This would provide a more up-to-date perspective on the topic.

**Response:** While restructuring the overall article, we have also included a few more relevant observations from studies between 2021-2024 in appropriate sections as advised in v2. 

**Comment 2: **Methodology: The methodology section could be expanded to include a more detailed description of the inclusion and exclusion criteria for the reviewed documents. This would improve transparency and allow for easier replication by other researchers.

**Response:** We have attempted to restructure the overall methodology to provide more clarity by expanding the inclusion and exclusion criteria for the reviewed documents.  

**Comment 3: **Data Availability: Ensure that all supplementary materials and data mentioned in the article are accessible to readers. This could involve providing direct links to the datasets or ensuring that they are available in an open-access repository.

**Response: **All supplementary materials and data mentioned in the article are accessible to readers through a digital repository. The accessible link is included in the data availability statement.

**Comment 4: **Citations: Review and update citations to include more recent and relevant studies. This will strengthen the article’s foundation and provide a more current context for the discussion.

**Response: **All relevant studies, including the newly added ones between 2021-2024, have been cited and referenced.

**Comment 5: **Discussion and Implications: The discussion could benefit from a more in-depth analysis of the potential challenges and limitations of implementing CBE in architectural education. Addressing these issues would provide a more balanced view and prepare educators for potential obstacles.

**Response: **The overall discussion section has been restructured to include extended discussion on challenges and a separate segment on implications, where we have attempted to provide a more in-depth analysis of the potential challenges and limitations of implementing CBE.

**Comment 6: **Clarify the criteria for document inclusion and exclusion in the narrative review.

**Response: **We have attempted to restructure the overall methodology to provide more clarity by expanding the inclusion and exclusion criteria.  

**Comment 7: **Ensure all supplementary materials are accessible and clearly referenced in the article.

**Response: **All supplementary materials and data mentioned in the article are accessible to readers through a digital repository (figshare).

**Comment 8: **Update the literature review with more recent studies to reflect current trends and practices.

**Response: **Updated.

 We have tried to address the concerns you have shared. Hopefully it would meet approval.

 Regards.

# Reviewer report: Reviewer response for version 1

**DOI:** 10.5256/f1000research.162950.r325164

**Published:** 2024-09-26

**Authors:** Jayendira P Sankar

**Affiliations:** 1 Master of Business Administration, University of Technology Bahrain, College of Administrative and Financial Sciences, Salmabad, Bahrain

**Relevance of Competency Based Education for Architectural Education in India**

**Dear Editor,**

 Thank you for giving me the opportunity to review this article. While the article appears complete,My initial question is whether it should be classified as a research article or a review article. Based on my understanding, it falls under the category of a review article, though the final classification is determined by the platform's guidelines.

 1. The details provided in the introduction, background, methodology, and results sections are acceptable. However, there is a need to separate the discussion, implications, and suggestions for future studies into distinct sections.

 2. While it is understood that the study's timeline covers the period from 2000 to 2021, the authors could strengthen their findings and implications by including relevant studies from 2022 to 2024. This would help provide more up-to-date support for the conclusions.

**Kindly follow the structure.**

 1. Introduction- provides background, states the research's purpose, discusses previous research leading up to the study, and may state a hypothesis or question.

 2. Literature review- describes the publication, a summary of the publication’s main points, a discussion of research gaps, and an evaluation of the publication’s contribution to the topic.

 3. Method or Methodology- describes how the research was conducted, with details about the study sample, assessment measures and procedure.

 4. Results or Findings- a summary of the findings presented in text or table format may have individual sections with specific information.

 5. Discussion, Comments or Conclusion-explains how the results answered the research question and may suggest future areas for research.

 6. References- a listing of works cited by the author(s).

 In my opinion, the article already contains sufficient information. The author(s) simply need to organize it more effectively to enhance its overall appeal.

 I request the author(s) to address the above comment.

 The editor may accept the article for indexing, provided that the aforementioned comments are adequately addressed.

**All the best**

**Thanks, and Regards**

Is the work clearly and accurately presented and does it cite the current literature?

Partly

If applicable, is the statistical analysis and its interpretation appropriate?

Partly

Are all the source data underlying the results available to ensure full reproducibility?

Partly

Is the study design appropriate and is the work technically sound?

Partly

Are the conclusions drawn adequately supported by the results?

Partly

Are sufficient details of methods and analysis provided to allow replication by others?

Partly

Reviewer Expertise:

The current research interests include Development Economics: economic growth, poverty, inequality, market failure, fiscal, economic and social condition, healthcare, education, employment sector, industrial and social infrastructure; Human Resource Management: employee welfare, work-family balance, consumer behaviour, retail marketing, corporate social responsibility, sustainability, and teaching-learning and assessments.

I confirm that I have read this submission and believe that I have an appropriate level of expertise to confirm that it is of an acceptable scientific standard, however I have significant reservations, as outlined above.

# Response

**Published:** 2024-10-30

**Authors:** Nikhil Kohale

**Affiliations:** MSAP, Manipal School of Architecture and Planning, MAHE, Manipal, Karnataka, India

**IN RESPONSE TO SUGGESTIONS/COMMENTS FROM REVIEWER 2** ****(Mr. Jeyendira P Sankar, Bahrain)

**Comment 1: **While the article appears complete, my initial question is whether it should be classified as a research article or a review article.

**Response:** Our intended research article has followed a narrative review approach. However, for clarity and based on the observation/ suggestion made, we have now formatted the same as a review article as per the suggested structure within the journal format.   

**Comment 2: **While it is understood that the study's timeline covers the period from 2000 to 2021, the authors could strengthen their findings and implications by including relevant studies from 2022 to 2024. This would help provide more up-to-date support for the conclusions.

**Response:** While restructuring the overall article, we have now included observations from relevant studies between 2021-2024 in appropriate sections as advised. 

**Comment 3: **Kindly follow the structure.

 1. Introduction- *provides background, states the research's purpose, discusses previous research leading up to the study, and may state a hypothesis or question.*

 2. Literature review- *describes the publication, a summary of the publication’s main points, a discussion of research gaps, and an evaluation of the publication’s contribution to the topic.*

 3. Method or Methodology- *describes how the research was conducted, with details about the study sample, assessment measures and procedure.*

 4. Results or Findings- *a summary of the findings presented in text or table format may have individual sections with specific information.*

 5. Discussion, Comments or Conclusion- *explains how the results answered the research question and may suggest future areas for research.*

 6. References- *a listing of works cited by the author(s).*

**Response:** We have now followed the Review template as provided by the Journal, and incorporated the advised structure. It can be seen in the update version in the overall document.

**Comment 4: **The article already contains sufficient information. The author(s) simply need to organize it more effectively to enhance its overall appeal.

**Response: **We have restructured and reorganized the overall content, while adding new findings from recent publications as well, to increase its overall appeal.

**Comment 5: **The editor may accept the article for indexing, provided that the aforementioned comments are adequately addressed.

**Response:** We hope that we have incorporated all comments in the overall narrative of the article as per your suggestion, for enhanced clarity and towards gaining approval.

# Reviewer report: Reviewer response for version 1

**DOI:** 10.5256/f1000research.162950.r307236

**Published:** 2024-08-19

**Authors:** Fadzidah Abdullah

**Affiliations:** 1 International Islamic University Malaysia, Kuala Lumpur, Federal Territory of Kuala Lumpur, Malaysia

1. The research is very good and comprehensive. it shows the researcher has done very deep investigation.

 2. However, the researcher did not really explain why there is a need to transfer from Content Based to competency based.  In fact, competency could NOT be earned in architecture school alone, it would be earned more during the practice. Whilst, content based is easier as we could complete the syllabus on time based basis. Another point,  experience in practice is very important to show one's competency. Meaning, investigating Competency could not be comprehensively investigated within the campus environment.

 3. The identified 8 themes may help  us to understand the current scenario, but each day there are new innovation that requires arch schools to move forward continuously. Something that is moving is very difficult to measure both  qualitatively and quantitatively.

 4. Hence, having this research regarded as narrative is  very smart because it is very difficult to draw a conclusion. Therefore, result would depends on one's interpretation.

 5. However, author has introduced a new term,"context based". This should not be the conclusion as if running away from giving solution to CBE.

 6. I would suggest the author to omit any mention of context based education.

Is the work clearly and accurately presented and does it cite the current literature?

Yes

If applicable, is the statistical analysis and its interpretation appropriate?

Partly

Are all the source data underlying the results available to ensure full reproducibility?

Yes

Is the study design appropriate and is the work technically sound?

Yes

Are the conclusions drawn adequately supported by the results?

No

Are sufficient details of methods and analysis provided to allow replication by others?

Yes

Reviewer Expertise:

architecture education

I confirm that I have read this submission and believe that I have an appropriate level of expertise to confirm that it is of an acceptable scientific standard, however I have significant reservations, as outlined above.

# Response

**Published:** 2024-10-30

**Authors:** Nikhil Kohale

**Affiliations:** MSAP, Manipal School of Architecture and Planning, MAHE, Manipal, Karnataka, India

**IN RESPONSE TO COMMENTS FROM REVIEWER 1 **(Mr. Fadzidah Abdullah, Malaysia)

**Comment 1:** Explain why there is a need to transfer from Content Based to competency based.

**Response: **Apart from the overall discussion, a brief explanatory text has been added in the introduction segment.

**Comment 2: **Author has introduced a new term, "context based". This should not be the conclusion as if running away from giving solution to CBE. I would suggest the author to omit any mention of context based education

**Response: **The term ‘context-based education’ has been omitted. Although, the word ‘context’ has been retained/ used as an explanation for a larger narrative supporting competency based education (CBE), wherever necessary.
